# First in man study: Bcl-Xl_42-CAF^®^09b vaccines in patients with locally advanced prostate cancer

**DOI:** 10.3389/fimmu.2023.1122977

**Published:** 2023-03-14

**Authors:** Sofie Kirial Mørk, Per Kongsted, Marie Christine Wulff Westergaard, Benedetta Albieri, Joachim Stoltenborg Granhøj, Marco Donia, Evelina Martinenaite, Morten Orebo Holmström, Kasper Madsen, Anders H. Kverneland, Julie Westerlin Kjeldsen, Rikke Boedker Holmstroem, Cathrine Lund Lorentzen, Nis Nørgaard, Lars Vibe Andreasen, Grith Krøyer Wood, Dennis Christensen, Michael Schantz Klausen, Sine Reker Hadrup, Per thor Straten, Mads Hald Andersen, Inge Marie Svane

**Affiliations:** ^1^ Department of Oncology, National Center for Cancer Immune Therapy (CCIT-DK), Copenhagen University Hospital, Herlev, Denmark; ^2^ IO Biotech Aps, Copenhagen, Denmark; ^3^ Department of Immunology and Microbiology, Faculty of Health and Medical Sciences, University of Copenhagen, Copenhagen, Denmark; ^4^ Department of Urology, Copenhagen University Hospital, Herlev, Denmark; ^5^ Statens Serum Institut, Center for Vaccine Research, Copenhagen, Denmark; ^6^ EVAXION BIOTECH A/S, Hørsholm, Denmark; ^7^ Department of Health Technology, Technical University of Denmark (DTU), HEALTH TECH, Kongens Lyngby, Denmark

**Keywords:** prostate cancer, peptide, immune response, cancer vaccine, immunotherapy

## Abstract

**Background:**

The B-cell lymphoma-extra-large (Bcl-XL) protein plays an important role in cancer cells’ resistance to apoptosis. Pre-clinical studies have shown that vaccination with Bcl-XL-derived peptides can induce tumor-specific T cell responses that may lead to the elimination of cancer cells. Furthermore, pre-clinical studies of the novel adjuvant CAF^®^09b have shown that intraperitoneal (IP) injections of this adjuvant can improve the activation of the immune system. In this study, patients with hormone-sensitive prostate cancer (PC) received a vaccine consisting of Bcl-XL-peptide with CAF^®^09b as an adjuvant. The primary aim was to evaluate the tolerability and safety of IP and intramuscular (IM) administration, determine the optimal route of administration, and characterize vaccine immunogenicity.

**Patients and methods:**

Twenty patients were included. A total of six vaccinations were scheduled: in Group A (IM to IP injections), ten patients received three vaccines IM biweekly; after a three-week pause, patients then received three vaccines IP biweekly. In Group B (IP to IM injections), ten patients received IP vaccines first, followed by IM under a similar vaccination schedule. Safety was assessed by logging and evaluating adverse events (AE) according to Common Terminology Criteria for Adverse Events (CTCAE v. 4.0). Vaccines-induced immune responses were analyzed by Enzyme-Linked Immunospot and flow cytometry.

**Results:**

No serious AEs were reported. Although an increase in T cell response against the Bcl-XL-peptide was found in all patients, a larger proportion of patients in group B demonstrated earlier and stronger immune responses to the vaccine compared to patients in group A. Further, we demonstrated vaccine-induced immunity towards patient-specific CD4, and CD8 T cell epitopes embedded in Bcl-XL-peptide and an increase in CD4 and CD8 T cell activation markers CD107a and CD137 following vaccination. At a median follow-up of 21 months, no patients had experienced clinically significant disease progression.

**Conclusion:**

The Bcl-XL-peptide-CAF^®^09b vaccination was feasible and safe in patients with l hormone-sensitive PC. In addition, the vaccine was immunogenic and able to elicit CD4 and CD8 T cell responses with initial IP administration eliciting early and high levels of vaccine-specific responses in a higher number og patients.

**Clinical trial registration:**

https://clinicaltrials.gov, identifier NCT03412786.

## Introduction

Prostate cancer (PC) is the second most commonly diagnosed cancer in males and the fifth leading cause of cancer-related death among men worldwide (1). Treatment options for patients with metastatic disease are limited, and when standard hormone deprivation-based therapies fail, the prognosis is poor. New therapies are therefore sorely needed to improve treatment outcomes (2, 3).

The field of cancer immunotherapy has undergone remarkable improvements in recent years, with checkpoint inhibitors as the most notable treatment option effective in a wide variety of malignancies (4). Besides Sipuleucel-T, a therapeutic cancer vaccine approved for patients with metastatic castration-resistant prostate cancer (mCRPC) by the Food and Drug Administration (FDA) and European Medicines Agency (EMA) in 2010 and 2013 respectively, immunotherapy has been generally ineffective in PC.

Various immunotherapies are currently under investigation in different cancer types. One of the modalities is therapeutic vaccines which aim to amplify the number of tumor-specific T cells responses through immunization. High levels of tumor-specific and tumor-associated antigens are expressed in PC (5) and T cells specific for several prostate-specific or -associated antigens can be found in the peripheral blood of patients with PC, suggesting that therapeutic vaccines may boost the prostate-cancer-specific T cell immunity (6, 7).

Peptide-based cancer vaccines usually consist of a amino acid (AA) sequence derived from tumor-specific or tumor-associated antigens (TAA). TAA can be found in healthy cells as well, but at elevated levels in cancer. For peptide cancer vaccines to be effective, they must contain both CD8+ and CD4+ epitopes to activate cytotoxic T lymphocytes (CTL) anti-tumor immunity and T helper cell activation to sustain the CTLs effector function (8).

The current study focuses on the anti-apoptotic protein Bcl-XL, which is overexpressed in PC (9). It has been suggested that an increase in Bcl-XL expression correlates with the lack of response to conventional chemotherapy and poor prognosis (10). Pre-clinical studies have shown that a naturally occurring T cell response against epitopes derived from the Bcl-XL protein exists in cancer patients. Furthermore, such T cells can directly kill cancer cells overexpressing Bcl-XL (11–14). Inhibition of Bcl-XL has been shown to restore the apoptotic process, which sensitizes the neoplastic cells to chemotherapy and radiotherapy. In contrast, high levels of Bcl-XL expression result in multi-drug resistance (15).

The peptide Bcl-XL_42 used in this study is a 42 AA long peptide derived from the Bcl-XL-protein sequence, which contain multiple *in silico* predicted epitopes for cytotoxic T cells (CD8+) and T helper cells (CD4+) across multiple tissue types (11, 13). A long peptide was selected because it has several advantages over short peptides (16). A long peptide cannot bind directly to major histocompatibility complex I (MHC-I) but must be taken up and processed by the antigen-presenting cells (APC) before being presented (17). Thus, using long peptide epitopes ensures that APCs take up the long peptide and stimulate both CD4+ helper T cells and CD8+ T cells, thereby inducing a more robust and more diverse immune response (18), which has been confirmed in animal models (18–21). Shorter peptides (8-10 AA) are restricted by HLA Class I molecules due to the short length not allowing the diversity required for the HLA polymorphism in the general population (22, 23). In contrast, the overlapping peptide epitopes on the long peptide diminish HLA-allotype restrictions. On the other hand, short peptides can bypass cross-presentation by binding directly to the MHC-I molecule but generally show an insufficient ability to induce help from the CD4+ T cells (24). Further, shorter peptides tend to induce immunological tolerance against the immunizing antigens because of outside loading *in vivo* of MHC class I, including B and T cells (17). This leads to immunological tolerance because B and T cells lack the co-stimulatory properties required for induction of an appropriate cytotoxic T cell response (25, 26).

The Bcl-XL_42 peptide was administered in CAF^®^09b, a novel liposome-based vaccine adjuvant. CAF^®^09b is based on the cationic surfactant dimethyl-dioctadecyl ammonium (DDA) in combination with two immune-stimulatory components: The C-type lectin receptor MINCLE agonist monomycoloyl glycerol (MMG) and the Toll-like receptor (TLR)-3 agonist Poly I:C. This adjuvant has, in pre-clinical models, shown superior ability to skew the immune response towards a type I/cytotoxic CD8+ T cell response, particularly when administrated IP compared to the traditional IM and subcutaneous (SC) administration routes (27–30). CAF^®^09b increases the uptake of peptides by APCs and activates the APCs to induce cross-presentation and -licensing, as well as proinflammatory signaling leading to activation of vaccine-specific CD4+ and CD8+ T cells (29).

Here we conducted a first in man phase I study in patients with locally advanced PC investigating the safety and immunogenicity of a vaccine comprising Bcl-XL_42 peptide and CAF^®^09b administered by different administration routes as IM injection would be more feasible than IP for future application.

## Patients and methods

### Patients

Eligible patients were ≥ 18 years. They had histologically confirmed adenocarcinoma of the prostate. In addition, patients had begun or were scheduled to start endocrine therapy with bicalutamide for either locally advanced PC or biochemical recurrence following curative therapy. Additional inclusion criteria were an Eastern Cooperative Oncology Group (ECOG) performance status of ≤2; and adequate function of vital organs. Critical exclusion criteria included: known or suspected severe autoimmune disease; history of severe allergy or anaphylactic reactions; bone or visceral metastases; treatment with immune suppressors such as corticosteroid or methotrexate; other malignant disorders within the last three years excluding planocellular and basocellular skin carcinoma; and previous treatment with other cancer vaccines.

### Trial design and treatment

The study was designed as a clinical first-in-man phase I trial. It was conducted at the National Center for Cancer Immune Therapy (CCIT-DK) and the Department of Oncology, Copenhagen University Hospital, Herlev, Denmark. The study protocol was approved by the Danish Medicines Agency as well as the Ethics Committee. The trial adhered to the Helsinki Declaration (31) and guidelines for Good Clinical Practice (GCP) (32). The trial was registered at clinicaltrials.gov (NCT03412786) and clinicaltrialsregister.eu (EudraCT No. 2015-003719-39). All patients provided a written informed consent form before inclusion. The primary endpoint was safety based on the occurrence of AE following the NCI Common Terminology Criteria for Adverse Events (CTCAE version 4.0). The secondary endpoint was immunogenicity of the vaccine and tertiary to investigate the difference in immune response related to the route of vaccine administration (IM vs. IP).

**Figure 1:**
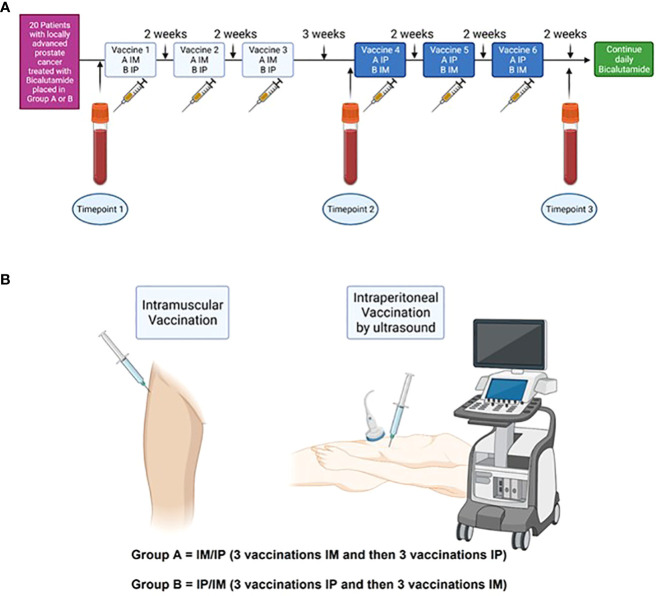
Study timelines **(A)** 20 patients with locally advanced prostate cancer were included and divided into two groups. Group A received three vaccines IM and then three vaccines IP, whereas Group B received the vaccines vice versa. At baseline, blood samples were collected (TP1). Treatment with Bcl_XL_42-CAF^®^09b vaccine was initiated shortly after and administered every second week for a total of 6 vaccinations, except between vaccine three and four, where the patient had a three weeks break. Blood samples were collected after three vaccines (TP2) and after six vaccines (TP3). **(B)** IM vaccine was administered in the musculus gluteus medius, whereas IP administration was done by ultrasound.

Twenty patients were planned for inclusion and treatment. The first ten patients were allocated to group A, and the latter ten patients to group B. Patients were assigned to receive a vaccination every second week for a total of 3 treatments followed by a three-week treatment-free interval before receiving the last three vaccines every second week *via* the alternate administration route. Thus, the first three vaccines were administered by IM injection and the last three vaccines by IP injections for group A. For group B, the administration routes were reversed (**Figures 1A, B**). Peripheral blood mononuclear cells (PBMCs) were collected at three time points: before injections (timepoint 1 – TP1), after three (timepoint 2 – TP2), and after six injections (timepoint 3 – TP3).

Assessments in the study included physical examination, ECOG performance, CTCAE grading, vital signs, and blood samples at every attendance to ensure safety.

### Bcl-XL-peptide, CAF^®^09b supply, and final vaccine formulation

Each vaccine was comprised of the Bcl-XL_42-peptide formulated with the CAF^®^09b adjuvant. The Bcl-XL_42-peptide consisted of the 42 AA long peptide:

(H-Val-Asp-Lys-Glu-Met-Gln-Val-Leu-Val-Ser-Arg-Ile-Ala-Ala-Trp-Met-Ala-Thr-Tyr-Leu-Asn-Asp-His-Leu-Glu-Pro-Trp-Ile-Gln-Glu-Asn-Gly-Gly-Trp-Asp-Thr-Phe-Val-Glu-Leu-Tyr-Gly-Oh).

Bcl-XL_42 (in total 50 μg/dose) was mixed in with the adjuvant (CAF^®^09b: 625 μg DDA/dose, 125 μg MMG/dose, 31 μg poly I:C/dose) immediately before administration to the patients. First, a 1.08 mL sterile filtered Tris reconstitution buffer was added to an ampule with 0.12 mL sterile filtered Bcl-XL_42-peptide and mixed. Afterwards, 1 mL of this peptide solution was added to a 2R vial containing 1.0 mL CAF^®^09b 2500/500/125. After thoroughly mixing, the final vaccine could be drawn into a syringe (dose-volume 0.5 ml).

The peptide was synthesized at purity ≥ 95% by Bachem Distribution Services GmbH, Switzerland, and delivered in lyophilized form. The peptide was stored at -20°C.

The CAF^®^09b adjuvant was manufactured by SSI, Denmark. The adjuvant was stored at +2-8°C.

### Blood samples and PBMC isolation

114 ml blood samples were collected three times during the study: Before vaccination (TP1), after three (TP2), and after six vaccinations (TP3). Peripheral blood mononuclear cells (PBMCs) were isolated with gradient-centrifugation using Lymphoprep (Takeda). Then cryopreserved in 90% human AB serum (HS) (Sigma.Aldrich, Ref. No H4522-100ml) and 10% dimethyl sulfoxide (DMSO) (WAK-chemie, Ref. No WAK-DMSO-10) using controlled-rate freezing (Cool-Cell, Biocision) in -80°C. PBMCs were kept at -140°C until use.

### Peptide pools

Minimal HLA class I (CD8+) binders were predicted *in silico* using Evaxion’s proprietary framework, a peptide:MHC prediction framework similar to NetMHC (doi: 10.1093/nar/gkaa379) or MHCflurry (doi: 10.1016/j.cels.2020.06.010). All peptides of length 8 to 12 were extracted from Bcl-XL_42 and predictions were generated for MHC allele specific binding. Predicted binders were divided into three patient-specific pools based on the binding prediction (**Supplementary Table 2a**) according to the twenty patients’ tissue types. Peptide binders predicted to be in the top 0.5 percentile (of a set of a million randomly sampled peptides) were added to Peptide Pool 1, binders scoring between 0.5 and 1 percentile in Peptide Pool 2, and binders scoring between 1 and 2 top percentiles were placed in Peptide Pool 3. HLA class II binders were predicted using the same framework, and overlapping predictions were merged into a total of 4 peptides and put in a single pool (Long Peptides pool) (**Supplementary Table 2b**). Overview of the different peptides and peptide groups can be found in **Supplementary Tables** (**Supplementary Tables 2a–c**).

### Pulsing and culturing of PBMCs

PBMCs were thawed, stimulated with Bcl-XL_42 and cultured in RPMIp/s + 10%HS (Gibco, Ref. no. 72400054), Penicillin-Streptomycin (pen/strep) (10,000U/mL) (Gibco, Ref. no 15140122) + 10% HS as described above. If needed, 1 ml of media was replaced with fresh culture media.

### IFN-gamma enzyme linked immunospot

To assess the immune response against Bcl-XL_42, we used *in vitro* IFN-gamma Enzyme Linked ImmunoSPOT (ELISPOT) assays on PBMCs described earlier (33). In short, PBMCs were thawed, counted, and rested for one hour. Then stimulated with 80 uM peptide (Bcl-XL_42) for two hours in 500μl media in a 24 well plate before the fresh medium was added, bringing the peptide concentration to 20 uM. The next day 120 U/ml IL-2 was added to the wells. The media was changed if it turned yellow during the *in vitro* stimulation. The cells were incubated for a total of 14 days in X-VIVO 15 (Lonza, Belgium, Ref. No LZ-BE02-060Q) supplemented with 5% human serum before re-stimulation with either Bcl-XL_42-peptide, peptide-pool-1, peptide-pool-2, peptide-pool-3, or long-peptides in the ELISPOT wells. Peptide-pool-1, -2, and -3 are patient-specific class I predicted minimal binders (HLA-A, -B, or -C), length 8 to 11 aa. Peptide pool-1 comprised the highest confidence predictions, pool-2 medium confidence predictions, and pool-3 the lowest confidence predictions. Long-peptides were patient-specific HLA-DRB1 (class II) predicted binders, merged into four consensus peptides.

DMSO was added in negative controls wells. Plates were incubated overnight at 37°C with 5% CO2 and developed according to the previously described protocol (33). The ELISPOT plates were analyzed using the Immunospot series 2.0 Analyzer (CTL, Shaker Heights, Ohio). The majority of samples in ELISPOT assays were set up in triplicates with a concentration of 2,1-3,0 x 10^5 cells/well, but for patient 12 (all timepoints), patients 13 (time point 1) and patient 14 (time point 1 and 2) the ELISPOT assays were performed in duplicates. The peptide-specific response was calculated by subtracting the mean spot count of negative control wells from the mean spot count in the peptide-stimulated wells.

Due to responses in patient 6 and patient 16 towards peptide-pool-3, we chose to do a similar analysis on PBMCs pre-stimulated with individual peptides in pool-3 and followed by 14 days of culture re-stimulation with peptide-pool-3 and the individual peptides (Patient 6: peptide 19, 21, 22, 24, 26, 28, 30 and Patient 16: peptide 19, 21, 23, 25, 27). All of these ELISPOT assays were performed in triplicates.

Peptide-specific ELISPOT responses were defined as valid if the difference between the control wells and the peptide stimulated wells was statistically significant according to the distribution-free resampling (DFR) rule (34). In cases where statistical analysis was not possible due to a limited number of replicates, responses were defined if the spot count in peptide stimulated wells was at least twice the count in control wells. Two patients had too numerous (spots) to count (TNTC: > 500 spots) why we could not perform the DFR test, but it was still defined as an actual immune response.

### Intracellular staining assay

After *in vitro* stimulation, PBMCs were harvested, counted and resuspended in an appropriate volume of culture media to a final concentration of 3x10^6 cells/ml. Cells were transferred to a 96 well plate in one of the following conditions: medium only, with Dimethyl Sulfoxide (DMSO) (WAK-chemie, Ref. no WAK-DMSO-10), 5µM Bcl-XL_42 peptide, or PMA (Sigma-Aldrich, Ref. no P1P1585-1MG)-Ionomycin (Sigma-Aldrich, Ref. no I3909-1ML), with a minimum of 2 wells per condition. The plate was incubated at 37°C and 5% CO_2_ for two hours, 50 µl Golgi solution (**Supplementary Table 3**) were added and cells were incubated for further 8 hours. Cell solutions were resuspended and pooled to one pre-labeled FACS tube per condition.

After two washing steps with Dulbecco’s Phosphate Buffered Saline (DPBS) (Gibco, Ref. no 14190250), cells were stained with live/dead stain (LIVE/DEAD™ Fixable Near-IR Dead Cell Stain Kit) (ThermoFisher Scientific, Ref. No L34976) following manufacturer instructions at 4°C for 10 minutes and for 20 minutes with 20 µl of extracellular antibodies stain (**Supplementary Table 3**).

Cells were washed with DPBS and incubated in the fridge overnight with 400 µl fixation/permeabilization solution (1:4 ratio, fixation/permeabilization concentrate (AH diagnostic, Ref. No 00-5123-43) and fixation/permeabilization diluent (AH Diagnostic, Ref. No 00-5223-56)).

The following day, permeabilization buffer 10X (AH Diagnostic, Ref. No 00-8333-56) was diluted 1:10 in sterile water and used for washing the cells twice. Cells were intracellularly stained with 20 µl antibody mix (**Supplementary Table 3**) and incubated at 4°C for 20-45 min. After a washing step with permeabilization buffer, each sample was resuspended in 100 µl of DPBS, and the samples were acquired with NovoCyte Quanteon Flow Cytometer. Obtained data were analyzed with FlowJo Software v10.6.1 using Boolean gating for identifying reactive CD4+ and CD8+ T cells subsets that were simultaneously positive to two out of the four investigated reactivity markers (CD107a, CD137, TNF-α, and IFN-γ). GraphPad Prism v9.0.0.121 was used for the graphical representation of data.

### CD107a and CD137 activation markers staining

Following *in vitro* stimulation, harvesting and plating of PBMCs was performed as described in the previous section. Anti-CD107a antibody (BS, Cat. No562623) was added to the culture and the cells were incubated at 37°C and 5% CO_2_ overnight. On the following day the cells were washed and stained extracellularly for 30 minutes at 4°C with live/dead stain and 20 µl antibody mix described in **Supplementary Table 3**. After incubation in the cold, cells were washed and 100 µl of DPBS. The samples were acquired with NovoCyte Quanteon Flow Cytometer and all cells positive to at least one marker between CD107a and CD137 were identified as being reactive. GraphPad Prism v9.0.0.121 was used for the graphical representation of data.

### Phenotyping of Peripheral Blood Mononuclear Cells using multicolor flow cytometry

Fluorochrome-labeled anti-human antibodies were used for surface staining of PBMCs (see **Supplementary Table 1** for details). The antibody mixtures additionally contained 10% Brilliant Violet Stain Buffer (BVSB)-plus (10X) (BD biosciences, Ref. No. 566385) and Dulbecco’s Phosphate-Buffered Saline (DPBS) (Gibco, Ref. No 14190250). PBMCs were thawed, washed with DPBS, stained with live/dead stain, and incubated in the dark at 4°C for 10 minutes.

Then, antibody mixtures were added, and the samples were further incubated in the dark at 4°C for 20 minutes. After staining, the cells were washed, resuspended in DPBS, and placed at 4°C until acquisition. Flow cytometry analysis was conducted on the Novocyte Quanteon Flow Cytometer (ACEA Biosciences) and analyzed using NovoExpress 1.4.1 software. An identical gating strategy was applied to the baseline and the follow-up samples. The gating strategy is shown in **Supplementary Figure 11**.

Blood samples for flow cytometry and blood cell counts were drawn simultaneously, and absolute counts were evaluated by aligning the lymphocyte count with CD3+, NK-cells, and B-cells during flow cytometry (35). Antibodies and gating strategies can be found in tables and **Supplementary Figures**.

### Statistical analysis

Clinical data were stored in a data repository hosted by OpenClinica (Waltham, MA, USA). ELISPOT responses were analyzed and determined using DFR method (34) using the statistical analysis program R version 3.6.1. IBM SPSS Statistics (Armonk, NY, USA) were used to show a clustered boxplot of outcome by different timepoints.

Statistical analyses were performed in GraphPad Prism V.9.2.0 (GraphPad Software, La Jolla, CA, USA). Wilcoxon matched-pairs signed rank t test was used to find the significance level in paired observations, and Mann-Whitney U test was used to compare ranks of unpaired observations. P values < 0.05 were considered significant.

## Results

### Patient characteristics

Between June 2018 and May 2020, 21 patients were included in the trial. One patient was excluded after two IM vaccinations due to lack of compliance. Twenty patients received all six vaccinations (Bcl-XL_42- CAF^®^09b). The median age at inclusion was 69,9 years (range 56 - 80 years), and the median time from diagnosis of PC to inclusion was 3.1 years (range 19 days to 11,5 years) (**Table 1**). Thirteen patients had undergone prior local therapy, five patients in group A and four in group B had undergone surgery (robot-assisted radical prostatectomy – RARP). Two patients in each group had received radiation therapy before vaccination (three had External Beam Radiation Therapy and one patient in group B had brachytherapy). All patients had begun standard treatment with bicalutamide before vaccination (the average time from initiation of bicalutamide to first vaccination was 70 days, ranging from 3 to 545 days). Median PSA at inclusion was 15.3 µg/L (range 0.1 - 96 µg/L). A summary of baseline patient characteristics is shown in **Table 1**.

Table 1Individual data for all patients in the trial.

**Table:** 

Patient number (Group A)	1	2	3	4	5	6	7	8	9	10
Age at inclusion	76	69	56	61	66	71	63	72	58	78
Time from diagnosis to inclusion (days)	419	3710	170	183	93	3273	661	3480	1491	19
Duration of treatment with Bicalutamid before inclusion (days)	407	533	6	3	68	4	0	4	3	3
Prior local therapy (yes/no)	no	yes (EBRT)	yes (RARP)	yes (RARP)	no	yes (EBRT)	yes (RARP)	yes (RARP)	yes (RARP)	no
TNM (M0 for all patients)	cT3Nx	T3N0	pT2aN1	pT3bN1	cT3bN1	cT3N0	pT3aN1	pT2cN0	pT3aN0	cT2/cT3Nx
PSA at inclusion	7,9	0,3	6,6	0,1	24	4,3	0,6	1,6	1,5	78
PSA after 3 vaccines	5,7	0,4	2,9	0,1	11	0,8	0,1	0,1	0,1	5,1
PSA after 6 vaccines	6,5	0,6	1,6	0,1	9,3	0,11	0,1	0,09	0	3
PSA at follow up	8,8	1,3	0,59	0,2	17	0,1	0,2	0,05	0,1	26
Time until follow up from last vaccination (days)	791	723	692	694	665	651	645	671	656	615
Time until follow up from first vaccination (days)	900	800	784	118	747	739	730	758	746	694

**Table:** 

Patient number (Group B)	11	12	13	14	15	16	17	18	19	20
Age at inclusion	77	71	76	66	71	80	75	73	72	67
Time from diagnosis to inclusion (days)	34	67	25	4069	4188	79	297	168	175	161
Duration of treatment with Bicalutamid before inclusion (days)	4	9	9	19	2	56	5	11	0	0
Prior surgery or radiation (yes/no)	no	no	no	yes (Brachy)	yes (EBRT)	no	yes (RARP)	yes (RARP)	yes (RARP)	yes (RARP)
TNM (M0 for all patients)	cT3aNx	cT3aN0	cT3aN0	cT2bNx	cT3aNx	cT3Nx	pT3aN0	pT3bN1	pT3aN1	pT3aN0
PSA at inclusion	13	96	46	1,5	5,2	4,5	7,8	0,31	1,5	4,8
PSA after 3 vaccines	0,1	34	3,4	0,2	2	3,1	0,1	0,05	0,09	0,3
PSA after 6 vaccines	0,09	23	1,8	0,1	1,2	2	0,05	0,05	0,09	0,2
PSA at follow up	0,05	1,2	0,6	0,01	0,4	1,4	0,5	0,05	0,1	0,2
Time until follow up from last vaccination (days)	491	532	538	518	483	325	377	407	248	154
Time until follow up from first vaccination (days)	575	616	620	594	562	399	464	484	325	231

### Adverse events and safety profile

All patients experienced either local or systemic AEs (**Table 2**). No vaccination-related AE > grade 2 were observed. The most common AEs were fatigue and injection site-specific reactions (pain at the injection site). Five patients experienced grade 1 fatigue after IP injections and three patients after IM injections. Eight patients experienced pain at the IP injection site, and one had a grade 2 reaction. Nine patients experienced pain at the IM injection site, and one had a grade 2 reaction. The most common systemic AE was flu-like symptoms in four patients (all had grade 1 - four patients after IP injection and two patients after IM injection). No severe (CTCAE ≥ grade 3) vaccination-related or systemic AEs were observed during the trial. A summary of AE and safety profiles is shown in **Table 2**.

**Table 2:** Adverse events registered during the trial.

Adverse event	Number of patients	Related to vaccination		Grade 1	Grade 2	Grade 3	IP	IM
		Possibly	Unrelated				Number of patients	
Abdominal pain	4	3	1	4	0	0	3	0
Constipation	2	2	0	2	0	0	2	0
Cough	3	0	3	3	0	0	0	0
Depression	1	0	1	1	0	0	0	0
Diarrhea	1	1	0	1	0	0	0	1
Dizziness	2	1	1	2	0	0	1	0
Dry Mucous Membranes	1	0	1	1	0	0	0	0
Dry Skin	2	1	1	2	0	0	1	0
Dygeusia	1	0	1	1	0	0	1	0
Dyspepsia	1	1	0	1	0	0	0	0
Fatigue	15	8	7	15	0	0	5	3
Fever	3	1	2	3	0	0	2	0
Flu Like Symtpms	4	4	0	4	0	0	4	2
Headache	1	0	1	1	0	0	0	0
Hepatobiliary disorder	1	0	1	1	0	0	0	0
Hyperhidrosis	2	2	0	2	0	0	2	2
Infection	3	0	3	3	0	0	0	0
Injection Site Reaction (pain)	14	14	0	12	2	0	8	9
Injection Site Reaction (redness)	1	1	0	1	0	0	0	1
Mucositis	1	0	1	1	0	0	0	0
Nightly thurst	1	1	0	1	0	0	1	0
Rash Maculo-papular	3	2	1	3	0	0	1	1
Tooth infection	1	0	1	1	0	0	0	0
Urinary tract infection	1	0	1	0	1	0	0	0

### Immune response to the vaccination antigen in peripheral blood

PBMCs were isolated at three time points to analyze immune responses against Bcl-XL by using IFN-gamma ELISPOT. In three patients, due to limited amount of PBMCs, the ELISPOT was performed in duplicates and not triplicates, why it was only possible to do statistical analysis on ELISPOT data from 17 patients (34).

**Figure 2:**
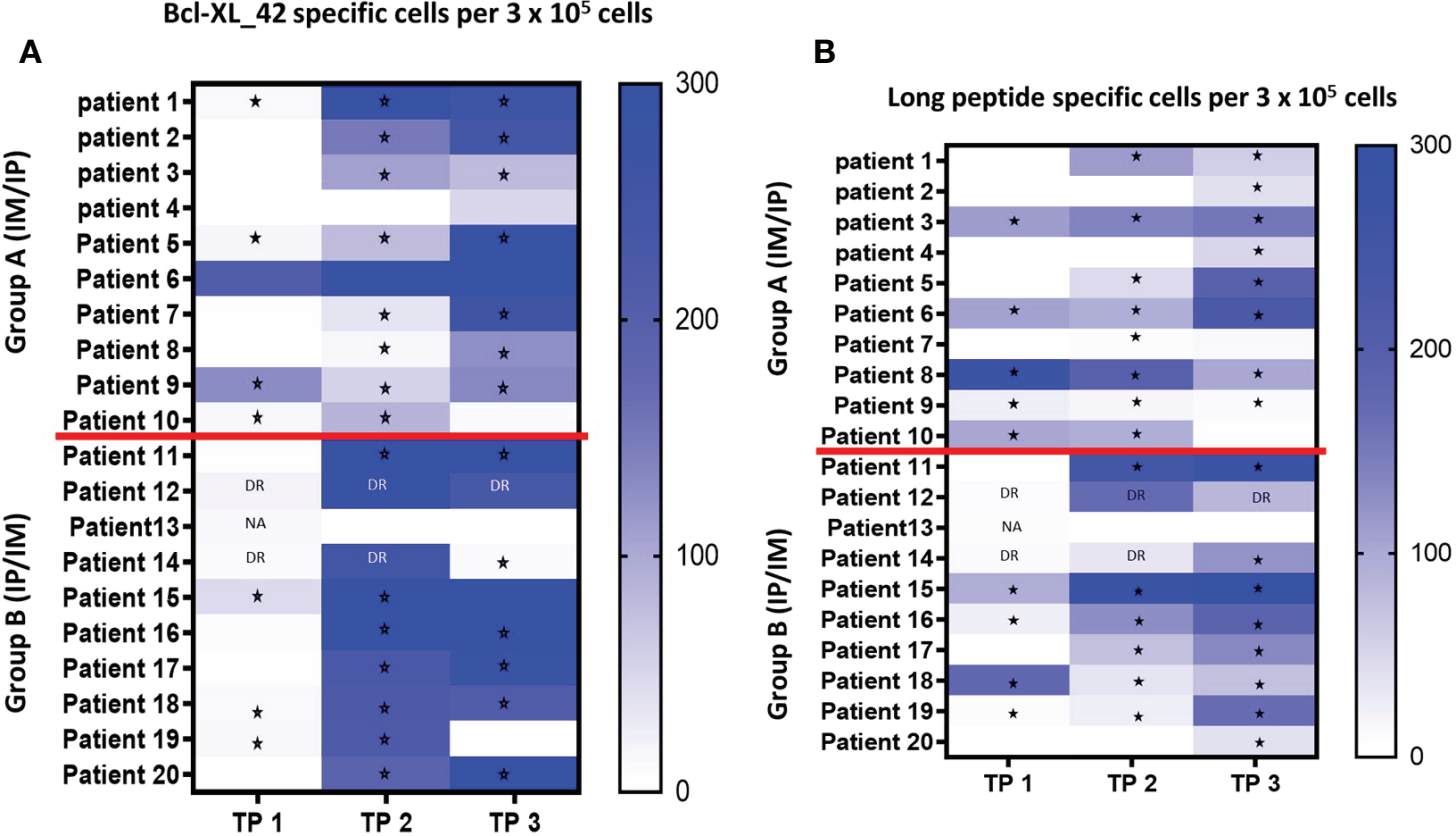
**(A, B)** Elispot analysis of PBMCs from all twenty patients at TP1 (before vaccination), TP2 (after three vaccinations), and TP3 (after six vaccinations). **(A)** PBMCs have been pre-stimulated with the vaccine peptide (BCL-XL_42) for 14 days before being restimulated with Bcl-XL_42, or **(B)** restimulated with the long peptide pool containing four HLA-class II *in silico* predicted peptides. Background spots were subtracted from the BCL-XL_42 wells. * P<0,05 statistically significant response based on DFR analysis. DR “Double Response” – the number of spots in peptide wells are 2x higher than control, cannot be statistically confirmed due to replicate number. NA Not able to do statistics due to duplicate and not triplicate. TNTC is too numerous to count (> 500). Patient 12 (all TPs), 13 (TP1), and 14 (TP1 and TP2) were not included because the ELISPOT were done in duplicates, and therefore it was not possible to do statistical analysis.

**Figure 3:**
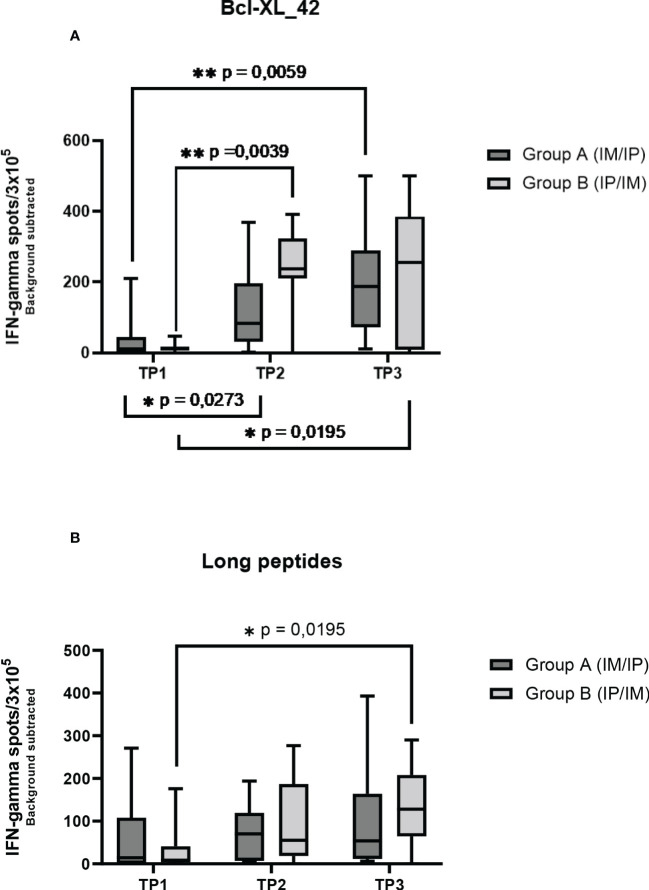
ELISPOT responses on PBMCs at three time points in two patient groups. Group A received three vaccinations IM first and then IP. Group B received accinations IP first and then IM. **(A)** the Elispot wells have been restimulated by the long peptide Bcl-XL_42, **(B)** and the HLA clas II predicted long peptide pool. Error bars depict the standard error of the mean. Statistical testing was performed using Wilcoxon matched pairs signed rank t-test. The stars are intended to flag levels of significance. If a p-value is less than 0.05, it is flagged with one star (*). If a p-value is less than 0.01, it is flagged with 2 stars (**).

**Figure 4:**
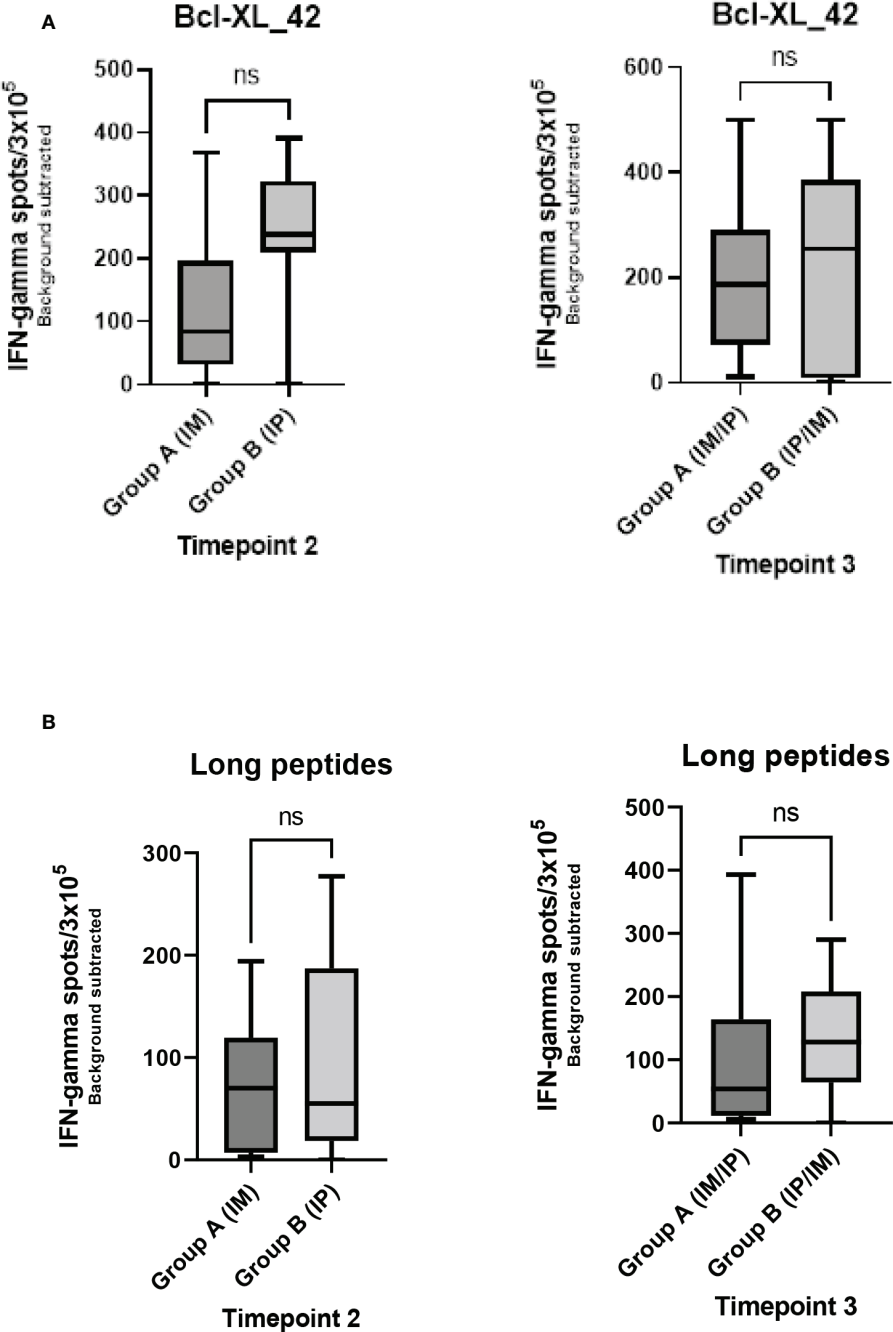
Comparison of Elispot data between group A and B at TP2 and TP3 for either stimulation with **(A)** the vaccination peptide (Bcl-XL-42) or **(B)** HLA class II predicted long peptide pool. Mann-Whitney U test was used to compare ranks of unpaired observations. No significant changes were found between groups A and B at any time points. ns meaning not significant.

Seven out of the 17 patients harbored Bcl-XL_42 baseline (TP1) reactivity (**Figure 2A**). In group A (IM to IP), nine of 10 patients displayed a significant response after the initial three vaccinations IM (TP2), and eight had a substantial reaction after the following three vaccinations IP (TP3). In seven patients in group A, the response amplitude increased between TP2 and TP3 (**Figure 2A**). Collectively for patient group A we identified a significant increase from TP1 to TP2 and TP1 to TP3 in Bcl-XL_42 specific ELISPOT responses (**Figure 3A**). In group B (IP to IM), seven out of eight patients showed significant response after the three initial vaccinations IP (**Figure 2A**). Seven patients had a significant response after the subsequent three IM vaccinations (**Figure 2A**). For four patients, the response amplitude decreased from TP2 to TP3, whereas for another four patients, it increased (**Figure 2A**). In group B, we also saw a significant increase in responses from TP1 to TP2 and TP1 to TP3 (**Figure 3A**). Overall, more patients in group B achieved a high vaccine response at TP2 compared to group A (IM), indicating that the IP administration route led to more potent vaccine responses than the IM route (**Figure 3A**). However, we observed no statistically significant difference in median reactivity level comparing ELISPOT responses towards Bcl-XL_42 peptide between group A and B at TP2 and TP3 (**Figure 4A**).

### Bcl-XL_42 HLA class I and II epitope specific immune responses

To investigate which of the many epitopes within the 42 aa long vaccine peptides was responsible for the T cell reactions, different peptide pools were created for ELISPOT analysis. The peptide pools were patient specific, based on their individual tissue type (**Supplementary Tables 2a–c**). Four peptide pools were predicted *in silico.* Three pools were HLA class I predicted (pool 1, 2 and 3), containing short peptides (**Supplementary 2a, b**), and one pool was HLA class II predicted, including four merged long peptides (long peptide pool) (**Supplementary Table 2c**). All peptides were embedded in the Bcl-XL_42 peptide sequence. The long peptide pool did generally induce a more potent T cell reaction compared with the shorter peptide pools (**Supplementary Figures 1A, B**). In group B, we observed a significant increase in T cell responses towards the long peptide pool from TP1 to TP3 (**Figures 2B**, **3B**). We found no statistically significant difference in median reactivity level comparing ELISPOT responses towards the long peptide pool between group A and B at TP2 and TP3 (**Figure 4B**).

In addition, 15 of 20 patients demonstrated a positive reactivity to at least one HLA class I peptide pool (short peptide pool 1, 2 and 3) at TP2 orTP3 (**Supplementary Figures 1A, B**). No association with route of vaccine administration was found. Two patients (6 and 16) with strong ELISPOT responses towards peptide pool 3 were selected for further analyses to identify the response to individual peptides from the peptide pool 3 (**Supplementary Figure 2**). We identified a significant immune response towards three (peptides 19, 22, and 24) and five (peptides 19, 21, 23, 25, and 27) of the specific peptides in Patient 6 and 16, respectively. For Patient 6, the response amplitude towards peptide 24 was approximately the same at all time points (TP). Still, we observed an amplitude increase against peptides 19 and 22, which were significant at TP3 (after all six vaccinations) (**Supplementary Figure 3A**), which indicates that the IP injection induces superior responses against some epitopes. The amplitude of ELISPOT responses was higher in patient 16. Noticeably, the T cell response towards peptide 23 indicated the presence of a peptide immune response before vaccination (**Supplementary Figure 3B**). The response towards peptide 21 increased throughout the trial. However, responses towards peptides 19 and 25 decreased from TP2 to TP3, again indicating a superiority in IP injections for some peptides.

**Figure 5:**
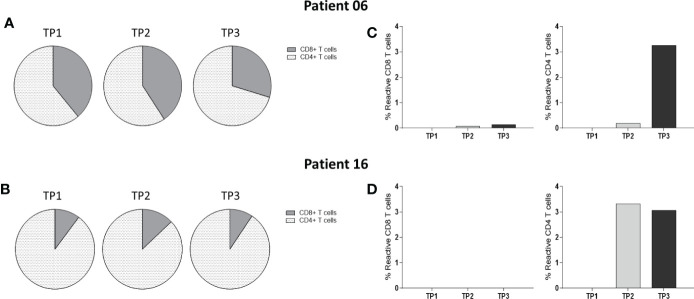
Multicytokines Intracellular staining assay of PBMCs from two patients at three TPs: Bcl-XL_42-specific reactivity of PBMCs isolated from two patients enrolled. Reactive cells were simultaneously positive to two of the four reactivity markers CD107a, TNFα, IFN-γ, and CD137. **(A, B)** Fractions of CD4+ and CD8+ T cells within PBMCs populations at each TP for the two patients. **(C)** Reactive CD4+ and CD8+ T cells were observed in a meager percentage in patient 6. **(D)** Reactive CD4+ T cells were observed in patient 16. Data were analyzed using FlowJo software and GraphPad Prism v9.

We further evaluated whether the Bcl-XL_42 T cell responses were dominated by CD4+ and/or CD8+ T cell reactivity by multicytokines intracellular staining assay (ICS). Bcl-XL_42-pre-stimulated PBMCs from patients 6 and 16 were analyzed for reactivity against Bcl-XL_42 in a T cell activation assay by flow cytometry (**Figure 5**). Peptide-specific CD4+ T cells were detected in both patients after vaccination. An increase from TP2 to TP3 was observed in patient 6 (**Figure 5C**), and a maximum response at TP2 in patient 16 (**Figure 5D**). No CD8+ reactivity was found in patient 16 at any timepoints (**Figure 5D**), whereas a slight increase in CD8+ T cell response was found at TP2 and TP3 in patient 6 (**Figure 5C**).

**Figure 6:**
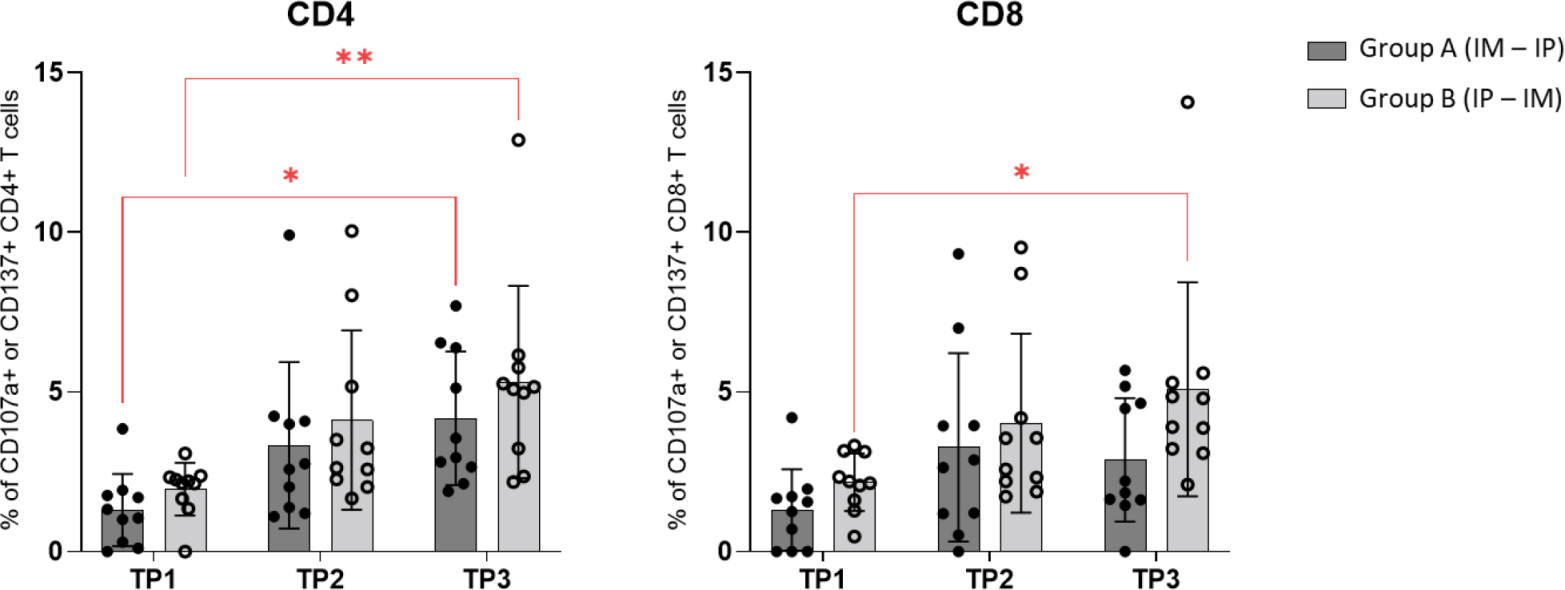
CD107a and CD137 activation markers staining: Comparison of CD4+ and CD8+ T cells expression of activation markers CD107a and CD137 in groups A and B at three timepoints by using Wilcoxon matched pairs signed rank t‐test.

By use of CD107a and CD137 activation markers staining on PBMCs we evaluated CD4 and CD8 T cell activation in group A and B at three timepoints (**Figure 6**). We found significant increase in activated CD4 T cells from TP 1 to TP2 in both group A and B. While activated CD8 T cells were only found to significantly increase in group B (IP/IM).

### Peripheral blood immune cell phenotype analyses throughout vaccination

Multicolor flow cytometry was performed on PBMCs to investigate different immune cell subtypes during Bcl_XL_42 vaccination.

In group A, the subpopulations of naïve and central memory (CM) T cells increased significantly during the treatment period (**Supplementary Figures 4A, B**), as did the percentage of PD1 and CD27 expressing CD8+ T cells (**Supplementary Figures 4C, D**). In group B, we also saw a significant increase in CM cells (**Supplementary Figure 4B**). In contrast a significant increase of CD4 regulatory T cells (Tregs) was only found in group A (**Supplementary Figure 4E**). The proportion of CD3+ T-cells out of total lymphocyte count increased significantly in group B from TP2 to TP3 (**Supplementary Figure 5A**). For group A the amount of NK cells out of total lymphocyte count decreased significantly during vaccination (**Supplementary Figure 5B**). At the same time, there was a significant increase in the fraction of B cells out of the total amount of lymphocytes in group A as well (**Supplementary Figure 5C**).

### Clinical disease course

All 20 patients remained on standard treatment with bicalutamide at the time of the last vaccine. Most patients had either decreasing or stable PSA values at a median follow-up of 20.9 months (ranging from 7.7 to 30 months after the first vaccination). At time of last follow-up (FU) none of the patients had initiated androgen depravation therapy (medical or surgical progression) underscoring a lack of clinically significant disease progression at this time. A decrease in PSA values was observed in 17 patients however most of these patients began bicalutamide shortly before inclusion. A slight PSA increase from inclusion until follow-up was observed in three patients. As a consequence, patient 5 and 10 had a 18F-sodium fluoride positron emission tomography - computed tomography (18F-NaF-PET-CT) performed revealing no metastases, and the patient remained on treatment with bicalutamide. Patient 1 also experienced an increase in PSA but because PSA levels had been known to fluctuate in this patient and there were no signs of clinical progression, this patient also remained on treatment with bicalutamide. A summary of the clinical disease course is shown in **Table 1**.

## Discussion

In this first-in-man study, 20 patients with hormone-sensitive PC were treated with a peptide vaccine containing the long peptide Bcl-XL_42 and the novel vaccine adjuvant CAF^®^09b. The vaccine was shown to be safe and tolerable. The most common side effect were mild injection site reactions (pain) and fatigue. Fatigue may result from concomitant bicalutamide treatment. The IP route of administration may cause transient discomfort and require specialized personnel, but still without need for specific medical intervention.

The low toxicity profile is in line with a phase I study from 2016 investigating the effects of therapeutic vaccination with short peptides from the proteins Bcl-2, Bcl-XL, and Mcl-1 in patients with relapse of multiple myeloma where no toxicity other than what was to be expected from standard treatment with bortezomib (proteasome inhibitor) were reported (36).

Pre-clinical evidence had shown CAF^®^09 to be a potent CD8+ T cell inducing adjuvant, with a significantly stronger CD8+ T cell response when administered IP compared with subcutaneous (SC) or IM (27, 29, 30, 37, 38). The principle of peptide cancer vaccines is based on selecting peptide sequences from either tumor-specific or tumor-associated antigens containing T cell epitopes, which are recognized by CD8+ and/or CD4+ T cells in a population with matching HLA haplotypes. T cell receptors recognize short linear AA sequences derived from an antigen, making it possible to use *in silico* bioinformatics as well as epitope mapping for prediction and selection of immunogenic aa sequences from a target tumor antigen (39, 40). In addition, when using overlapping or multi-epitope peptide sequences, issues such as diversity of HLA haplotypes, tumor heterogeneity, and tumor antigen downregulation may be possible to overcome.

By analyzing immune responses using ELISPOT we demonstrated significant Bcl-XL_42 specific T cell responses when vaccination was administered either IM/IP or IP/IM. Indeed, more patients had a potent Bcl-XL_42 response after initial IP injections. This supports the hypothesis of a better antigen presentation by the relevant APC subsets by this administration route (29). Importantly the IP injections did not lead to more side effects than IM. However, a potential concern for future treatment feasibility is that the IP route is more complicated and demanding to handle for the clinical staff.

Further, HLA class I and class II predicted peptide pools were established based on *in silico* prediction. The choice of the shorter peptides, derived from Bcl-XL_42, were based on the predicted ability to induce a CD8+ immune responses. It was possible to induce T cell responses against all predicted peptide pools, which suggests that the chosen Bcl-XL_42 peptide indeed include many different epitopes across the 42 aa sequence. Overall, 15 of the patients developed immune response to at least one HLA class I peptide pool at TP2/TP3. Significant peptide-specific responses were found in two patients examined. The responses in patient 6 (IM/IP) were less prominent than in patient 16 (IP/IM). However, two of the three peptide-specific responses were first induced at TP3 (after both IM and IP injection), whereas two of the responses in patient 16 peaked at TP2. Indicating that the IP injection could be superior for some epitopes. Summarized, ELISPOT analyses demonstrated immunization towards both CD8+ and CD4+ restricted epitopes comprised in Bcl-XL_42, showing that the 42 aa long peptide contains several T cell epitopes.

The vaccination with the long 42mer peptide induced strong T-cell responses in all patients, which suggested that the predicted short peptides were not the most immunogenic epitopes *in vivo*. However, the length of the vaccine peptide could have provided an obstacle for the processing and antigen presentation of some of these short peptides, which subsequently prevented these from being presented to the T-cells. In case of the latter, an alternative approach would be to include a combination of shorter epitopes, e.g., overlapping 20mer peptides into the vaccine.

To further determine whether the responses were CD4+ and/or CD8+ dominated, ICS was performed on Bcl-XL_42 stimulated PBMCs from patients 6 and 16 (**Figure 5**). Reactive CD4+ responses were found in both patients after vaccination. However, no CD8+ reactivity was detectable in patient 16 at any timepoints, whereas a small CD8+ response was found at TP2 and TP3 in patient 6. These findings do not coincide with the ELISPOT analyses where reactivity to short class I restricted Bcl-XL-peptides indeed was suggested. One explanation could be suboptimal processing and presentation of CD8+ epitopes in the ICS setup. Still, maybe the vaccine primarily activated CD4+ T cells. However, pre-clinical findings by Korsholm et al. (30) and Schmidt et al. (29), have previously demonstrated robust CD8+ T cell responses upon IP vaccination with recombinant, synthetic peptide and CAF^®^09 in mice. Further we demonstrated vaccination related increase in CD107a/CD137 activated CD4 T cells in both group A and B while increase in CD8 T cell activation was only demonstrated in group B. (**Figure 6**). Larger patient cohort studies are needed to further dissect differences in CD4 and CD8 T cell responses when vaccination are given either IM or IP.

Multicolor flow cytometry was performed on PBMCs to investigate the general immune cell phenotype development during Bcl-XL_42 vaccination. We observed an increase in PD1 positive CD8+ T cells from TP1 to TP3 in group A (**Supplementary Figure 4C**). PD1 is known to be upregulated on the cell surface of activated T cells (41), suggesting an increase in activation/antigen-experienced CD8+ T cells due to the vaccination in this study. In group A, we found the expression of CD27 on CD8+ T cells increased significantly across the vaccination (**Supplementary Figure 4D**), which indicates an activation of CD8+ T cells (42). This pathway is essential for sustained effector functions, T cell survival, and the development of memory T cells (43).

The two cohorts were relatively small, and the phenotyping changes observed in the peripheral immune cells during vaccination are not conclusive. Therefore, a more extensive cohort study is needed. Also, the patients in group 1 were significantly younger (Group A mean in age 67 years vs Group B mean in age 72,8 years), but we saw no difference in the responses induced. Neither did we find a correlation between PSA level at inclusion and immune responses.

Interestingly, none of the 20 patients treated in the study experienced clinically significant disease progression at a median FU of 20,91 months (**Table 1**). However, in a randomized study from 2002 comparing treatment with bicalutamide versus placebo, only 16.3% experienced disease progression in the bicalutamide arm at a median follow-up of 3 years (44). Only two patients out of twenty included surpassed the three years of bicalutamide treatment at the time of follow up. Thus, it is not unexpected that the majority of the included patients in our study still have stable PSA levels.

Our trial suffers from the limitations of a small population size of only twenty patients, no control group, and a non-randomized design. Future studies should include larger patient cohorts and maybe only one administration route (IP vs. IM) for each group. Also, it would be interesting to see if there were long-lasting immune responses at later follow-up time points. Earlier studies have shown that Bcl-XL expression can contribute to androgen resistance and thereby promote the progression of PC (9). Thus, for future studies, it would be interesting to investigate the Bcl-XL_42-CAF^®^09b in patients with increasing PSA during endocrine therapy.

## Conclusion

In conclusion, we demonstrated that the Bcl-XL_42-CAF^®^09b is safe and capable of eliciting CD4+ and CD8+ T cell responses both by IP and IM injections in a clinical setting where patients received concurrent antiandrogenic treatment. In addition, initial IP administration leads to an early and high increase in vaccine-specific immunity in more patients compared to IM injections.

## Data availability statement

The original contributions presented in the study are included in the article/**Supplementary Material**. Further inquiries can be directed to the corresponding author.

## Ethics statement

The studies involving human participants were reviewed and approved by National Videnskabsetisk Komité - Nationalt Center for Etik. The patients/participants provided their written informed consent to participate in this study. Written informed consent was obtained from the individual(s) for the publication of any potentially identifiable images or data included in this article.

## Author contributions

SM: Primary investigator, author of manuscript. PK: Early on, primary investigator, author of protocol. MW: Primary lab supervisor. BA: Lab work, ICS. JG: Lab work, Phenotyping. MD: Supervisor. EM: Lab work, Elispot. MH: Lab work, Elispot. KM: statistics. AK: Clinical wor. JK: Clinical work. RH: Clinical work. CL: Clinical work. NN: Clinical work. LA: Vaccine and adjuvant development. GW: Vaccine and adjuvant development. DC: Vaccine and adjuvant development. MK: AI prediction of peptides. SH: Project supervisor. PT: Project supervisor. MA: Project supervisor. IS: primary project supervisor. All authors contributed to the article and approved the submitted version.

## Supplementary material

Supplementary Figure 1
**(A)** Elispot analysis of PBMCs from Group A (IM/IP) individually at TP1 (before vaccination), TP2(after three vaccinations), and TP3 (after six vaccinations). PBMCs have been pre-stimulated with the 42 aa long peptide (BCL-XL_42), and re-stimulated with Bcl-XL_42, the three individual HLA class I predicted peptide pools (Pep-Pool 1, 2 and 3), and the four peptide-single pool (Long Peptides – HLA class II predicted). **(B)** Elispot analysis of PBMCs from Group B (IP/IM) individually. Background spots were subtracted from the BCL-XL_42 wells. * P<0,05 statistically significant response based on DFR analysis. NA = Not able to do statistics due to duplicate and not triplicate. Patient 12 (all TPs), 13 (TP1), and 14 (TP1 and TP2) were not included because the ELISPOT were done in duplicates, and therefore it was not possible to do statistical analysis.

Supplementary Figure 2 Elispot wells from patients 6 and 16. PBMCs had been prestimulated with Bcl-XL_42 peptide and restimulated with the vaccine-peptide and the three short peptide pools (HLA class I predicted), and the long peptide pool (HLA clas II predicted). HLA class I predicted peptide pool 3 in both patients showed interesting responses, and further analysis were done on PBMCs from the two patients from each patient group.

Supplementary Figure 3 Elispot analysis of PBMCs from **(A)** patient 6 and **(B)** patient 16. PBMCs have been prestimulated with Bcl-XL_42 and restimulated with the *in silico* HLA class I predicted peptide-pool-3 and the individual peptides for both patients, predicted by the patients individual tissue type.

Supplementary Figure 4 Phenotyping of Peripheral Blood Mononuclear Cells using multicolor flow cytometry. **(A)** Subpopulations of naïve T cells out of CD8+ cells, effector memory (EM) T cells out of CD4+ cells and CD8+ cells in group A at three TPs. **(B)** Subpopulation of central memory T cells out of CD8+ cells in group A and group B at three TPs. **(C)** Percentage of PD1 cells out of CD8+ cells in group A and B at three TPs. **(D)** Percentage of CD27 cells out of CD8+ cells in group A and B at three TPs. **(E)** Regulatory T cells (Treg) out of CD3+ T cells in group A and B at three TPs Statistical testing was performed using Wilcoxon matched pairs signed rank t-test.

Supplementary Figure 5 Phenotyping of Peripheral Blood Mononuclear Cells using multicolor flow cytometry. **(A)** Estimated CD3+ cell count out of total lymphocyte count in group A and group B at three TPs. **(B)** Estimated NK cell count out of total lymphocyte count in group A and group B at three TPs. **(C)** Estimated B cell count out of total lymphocyte count in group A and B at three TPs. **(D)** Percentage of single cells out of CD3+ cells in group A and B at three TPs Statistical testing was performed using Wilcoxon matched pairs signed rank t-test.

Supplementary Figure 6 Gating strategy myeloid cells.

Supplementary Figure 7 Gating strategy T cell diff.

Supplementary Table 1 Antibodies for extracellular staining. *10µl of a 1:100 dilution in DPBS were used.

Supplementary Table 2
**(A)** Division of three peptide pools including HLA class I predicted short peptides, Every pool is divided according to each patients specific tissue type. **(B)** HLA class I predicted peptide pools divided by rank. **(C)** HLA class II predicted peptide pool of long peptides. The predicted peptides were merged into a total of 4 peptides and put in a single pool (Long Peptides).

Supplementary Table 3 Reagents used for multicytokines intracellular staining assay and their specifications.

Supplementary Table 4 Tissue haplotype table.
