# Supplementary figures and images for: First in man study: Bcl-Xl_42-CAF®09b vaccines in patients with locally advanced prostate cancer
Source: Front Immunol. 2023 Mar 14;14:1122977. doi: 10.3389/fimmu.2023.1122977 (PMC10043415; doi:10.3389/fimmu.2023.1122977)

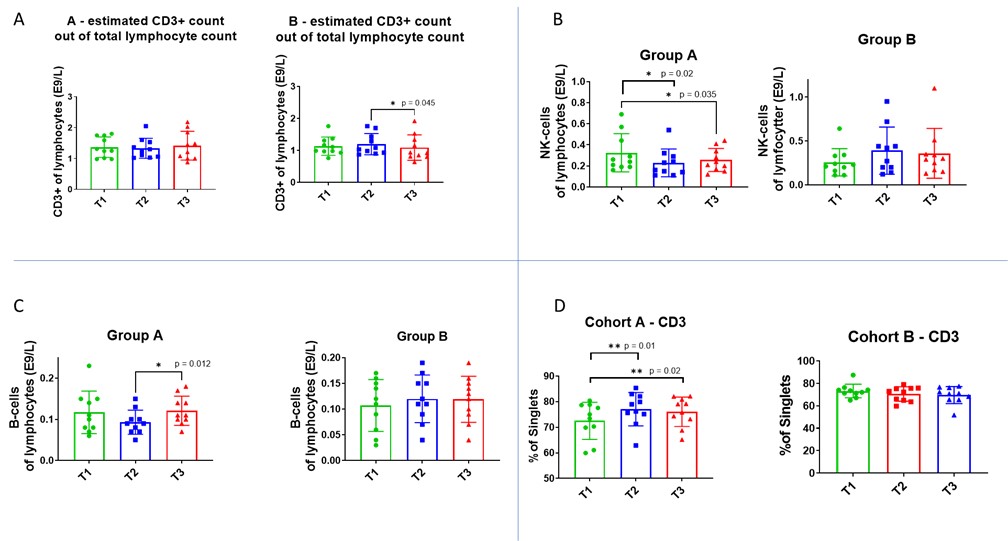

Supplement: Supplementary Figure 1 — (A) Elispot analysis of PBMCs from Group A (IM/IP) individually at TP1 (before vaccination), TP2(after three vaccinations), and TP3 (after six vaccinations). PBMCs have been pre-stimulated with the 42 aa long peptide (BCL-XL_42), and re-stimulated with Bcl-XL_42, the three individual HLA class I predicted peptide pools (Pep-Pool 1, 2 and 3), and the four peptide-single pool (Long Peptides – HLA class II predicted). (B) Elispot analysis of PBMCs from Group B (IP/IM) individually. Background spots were subtracted from the BCL-XL_42 wells. * P<0,05 statistically significant response based on DFR analysis. NA = Not able to do statistics due to duplicate and not triplicate. Patient 12 (all TPs), 13 (TP1), and 14 (TP1 and TP2) were not included because the ELISPOT were done in duplicates, and therefore it was not possible to do statistical analysis. [file Image_1.jpeg]

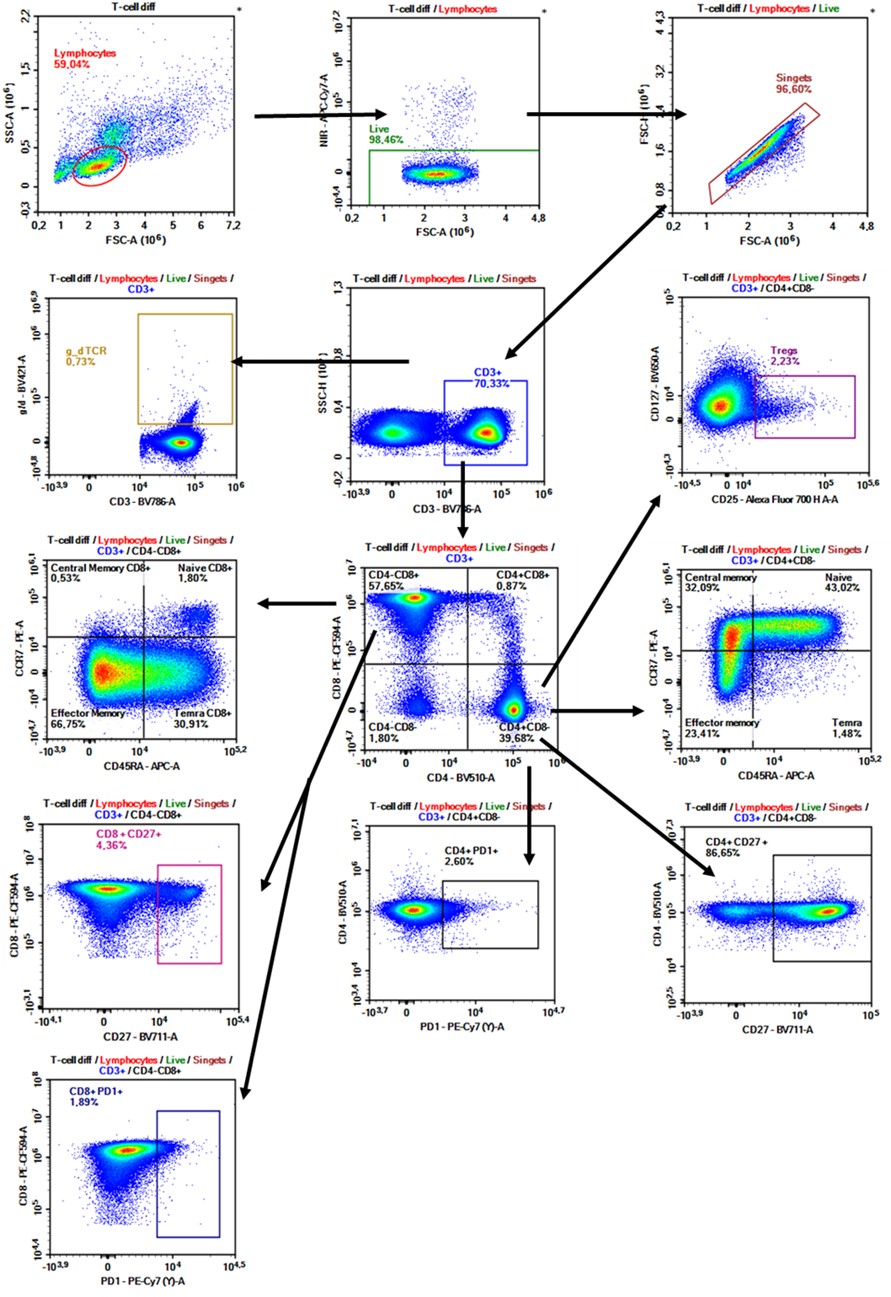

Supplement: Supplementary Figure 2 — Elispot wells from patients 6 and 16. PBMCs had been prestimulated with Bcl-XL_42 peptide and restimulated with the vaccine-peptide and the three short peptide pools (HLA class I predicted), and the long peptide pool (HLA clas II predicted). HLA class I predicted peptide pool 3 in both patients showed interesting responses, and further analysis were done on PBMCs from the two patients from each patient group. [file Image_2.jpeg]

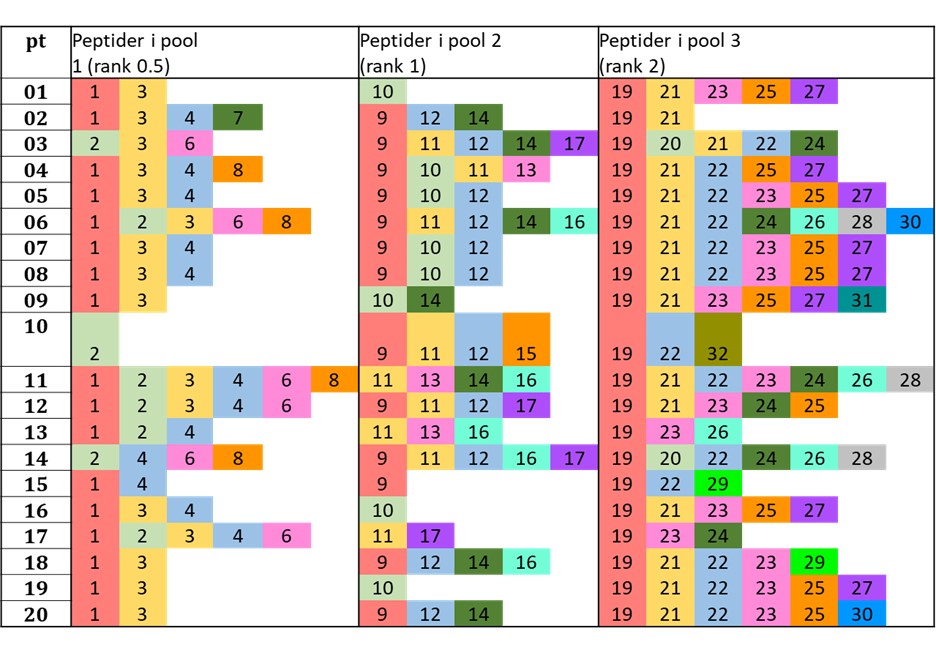

Supplement: Supplementary Figure 3 — Elispot analysis of PBMCs from (A) patient 6 and (B) patient 16. PBMCs have been prestimulated with Bcl-XL_42 and restimulated with the in silico HLA class I predicted peptide-pool-3 and the individual peptides for both patients, predicted by the patients individual tissue type. [file Image_3.jpeg]

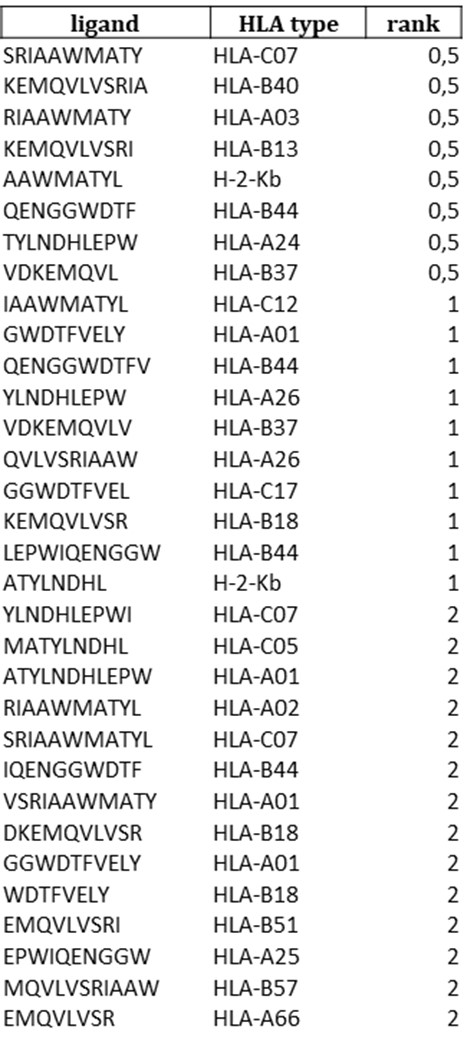

Supplement: Supplementary Figure 4 — Phenotyping of Peripheral Blood Mononuclear Cells using multicolor flow cytometry. (A) Subpopulations of naïve T cells out of CD8+ cells, effector memory (EM) T cells out of CD4+ cells and CD8+ cells in group A at three TPs. (B) Subpopulation of central memory T cells out of CD8+ cells in group A and group B at three TPs. (C) Percentage of PD1 cells out of CD8+ cells in group A and B at three TPs. (D) Percentage of CD27 cells out of CD8+ cells in group A and B at three TPs. (E) Regulatory T cells (Treg) out of CD3+ T cells in group A and B at three TPs Statistical testing was performed using Wilcoxon matched pairs signed rank t-test. [file Image_4.jpeg]

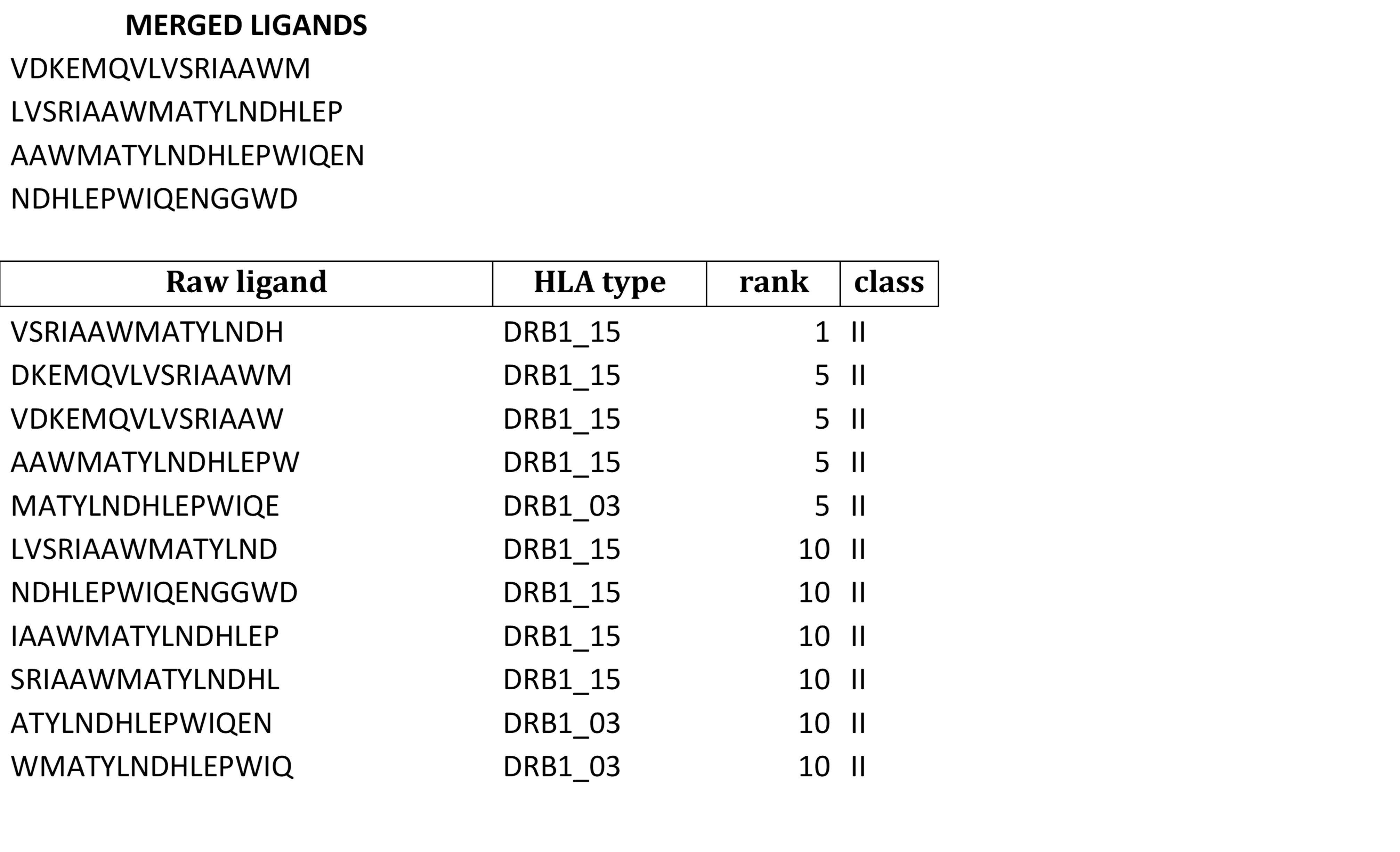

Supplement: Supplementary Figure 5 — Phenotyping of Peripheral Blood Mononuclear Cells using multicolor flow cytometry. (A) Estimated CD3+ cell count out of total lymphocyte count in group A and group B at three TPs. (B) Estimated NK cell count out of total lymphocyte count in group A and group B at three TPs. (C) Estimated B cell count out of total lymphocyte count in group A and B at three TPs. (D) Percentage of single cells out of CD3+ cells in group A and B at three TPs Statistical testing was performed using Wilcoxon matched pairs signed rank t-test. [file Image_5.jpeg]

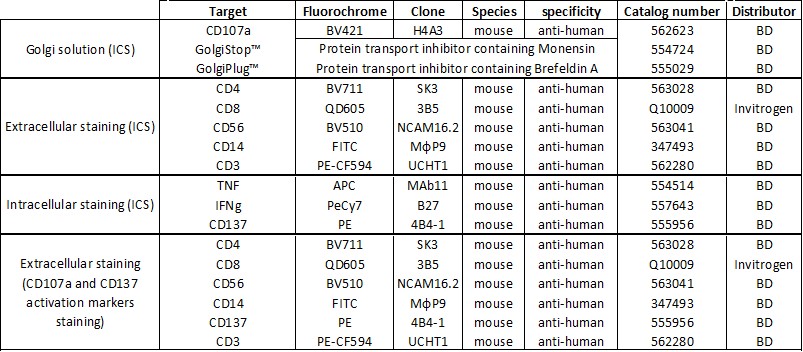

Supplement: Supplementary Figure 6 — Gating strategy myeloid cells. [file Image_6.jpeg]

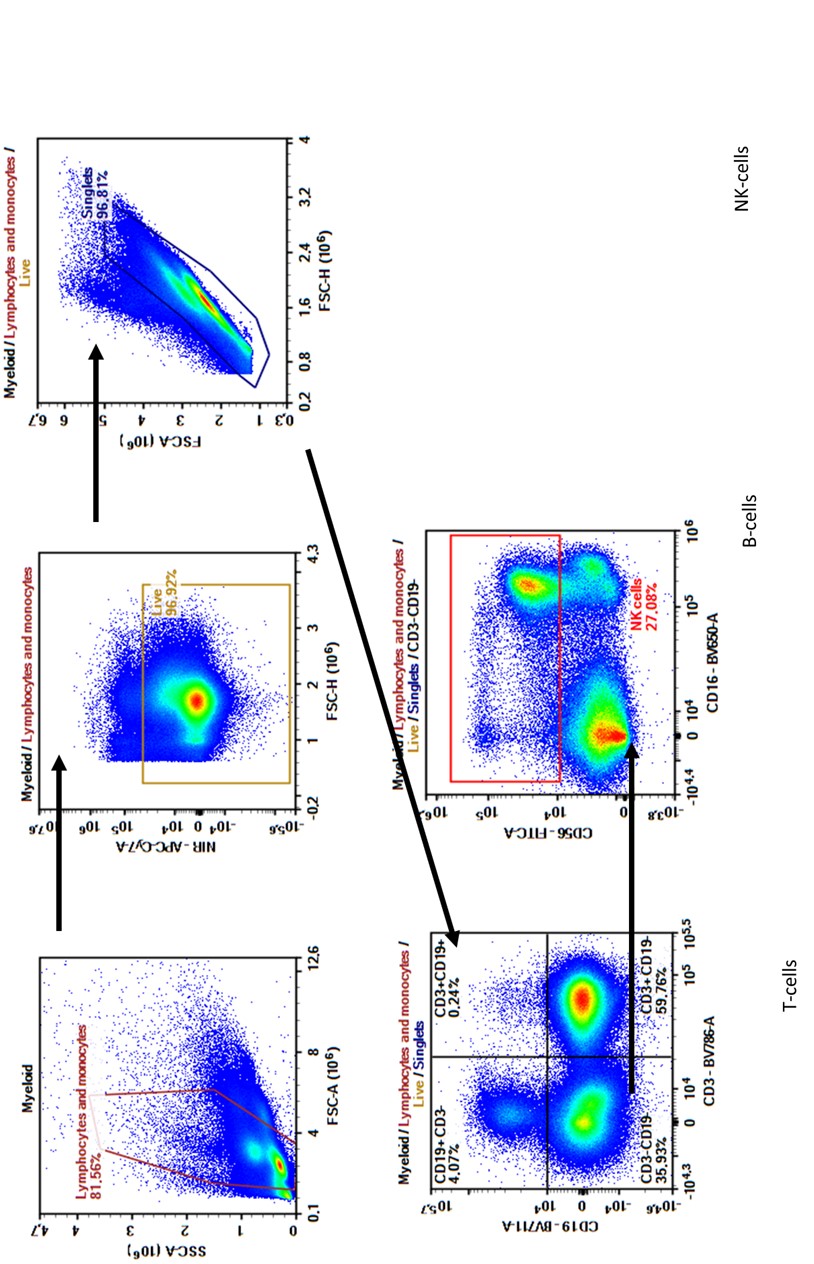

Supplement: Supplementary Figure 7 — Gating strategy T cell diff. [file Image_7.jpeg]

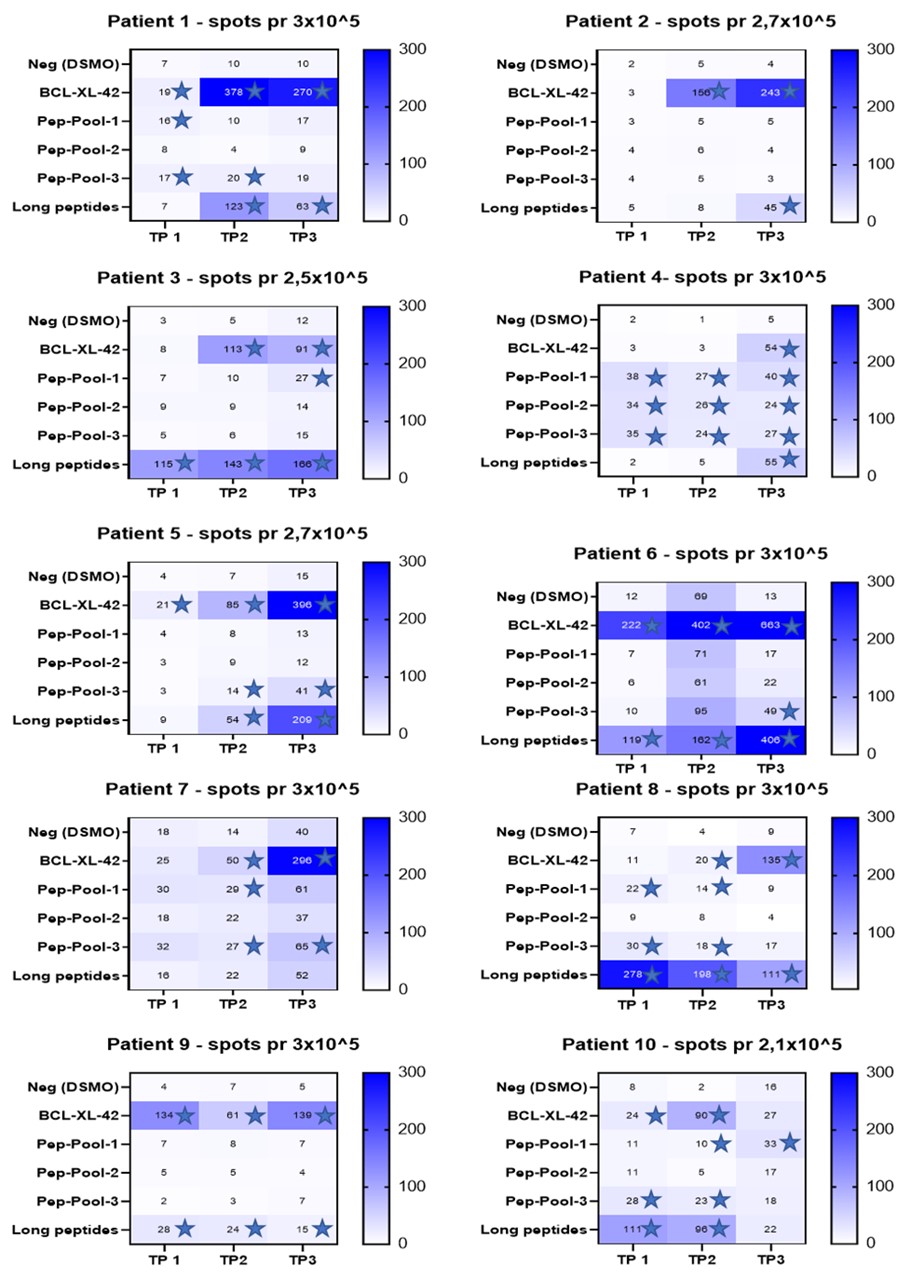

Supplement: Supplementary Table 1 — Antibodies for extracellular staining. *10µl of a 1:100 dilution in DPBS were used. [file Image_8.jpeg]

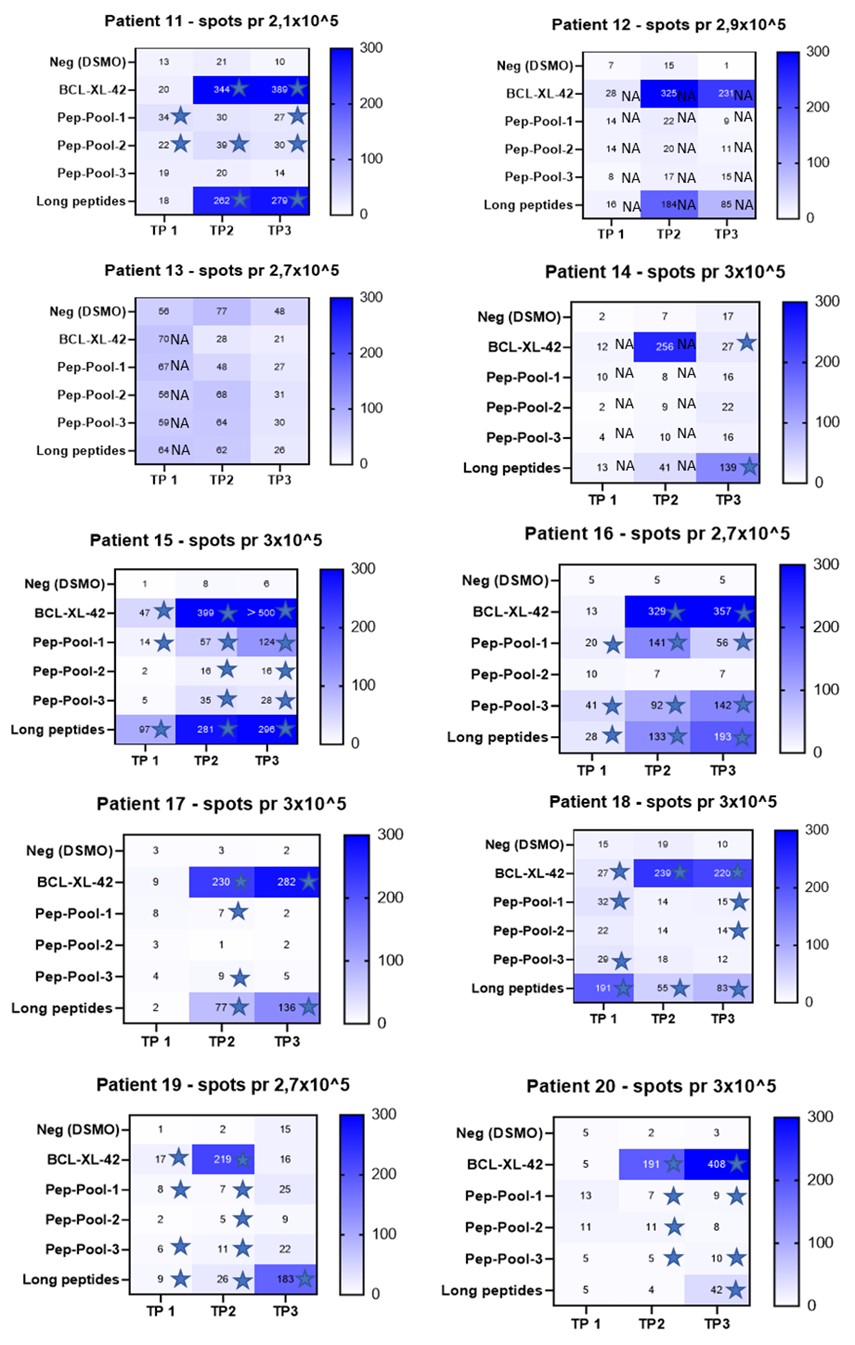

Supplement: Supplementary Table 2 — (A) Division of three peptide pools including HLA class I predicted short peptides, Every pool is divided according to each patients specific tissue type. (B) HLA class I predicted peptide pools divided by rank. (C) HLA class II predicted peptide pool of long peptides. The predicted peptides were merged into a total of 4 peptides and put in a single pool (Long Peptides). [file Image_9.jpeg]

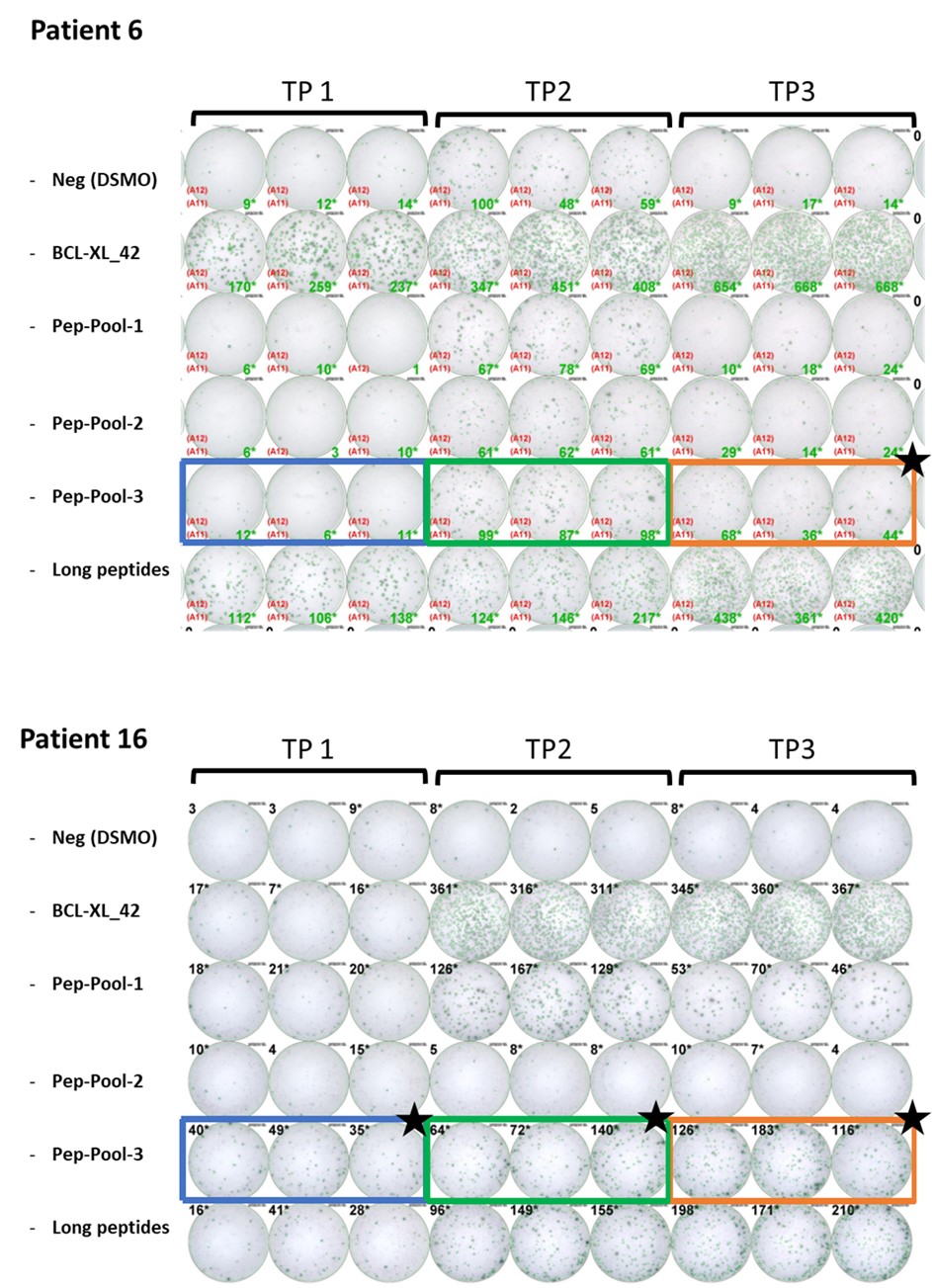

Supplement: Supplementary Table 3 — Reagents used for multicytokines intracellular staining assay and their specifications. [file Image_10.jpeg]

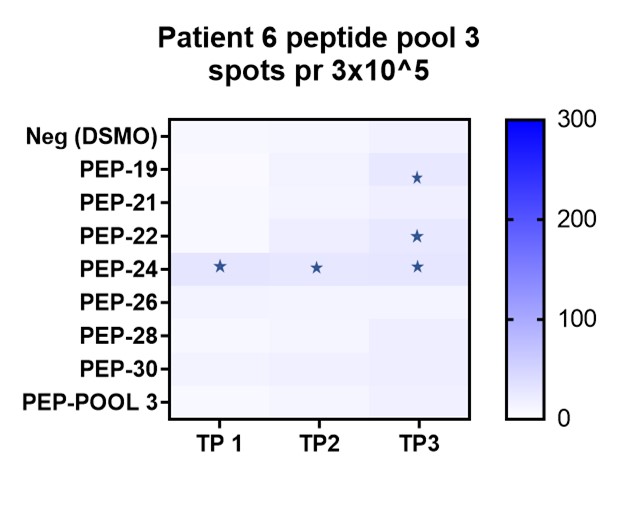

Supplement: Supplementary Table 4 — Tissue haplotype table. [file Image_11.jpeg]

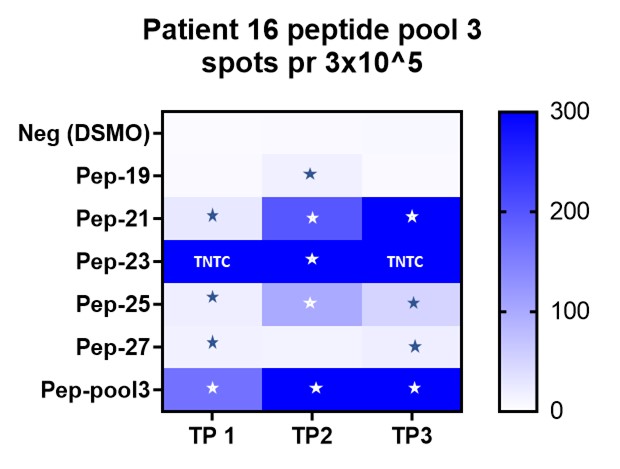

Supplement: Supplementary file 12 [file Image_12.jpeg]

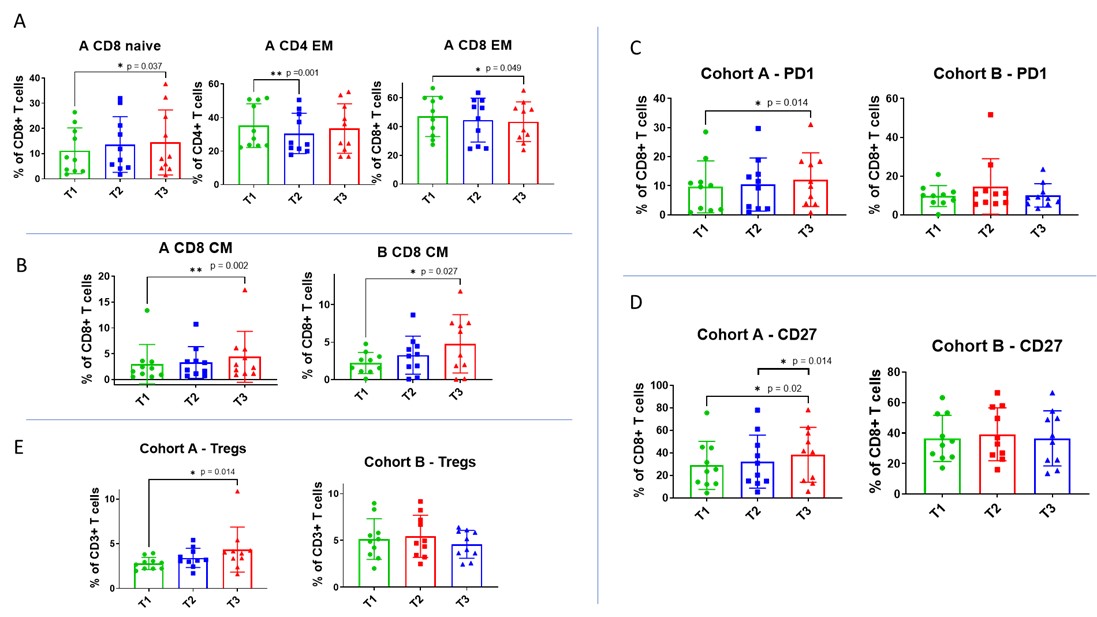

Supplement: Supplementary file 13 [file Image_13.jpeg]

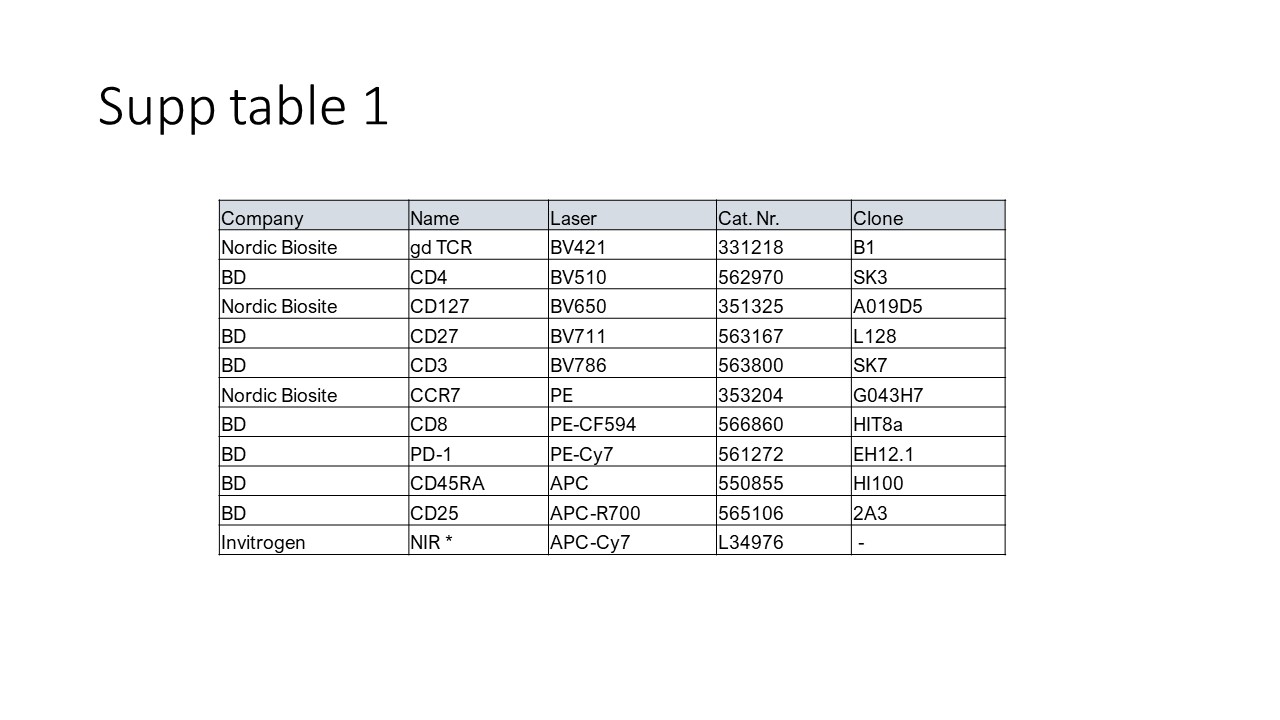

Supplement: Supplementary file 14 [file Image_14.jpeg]

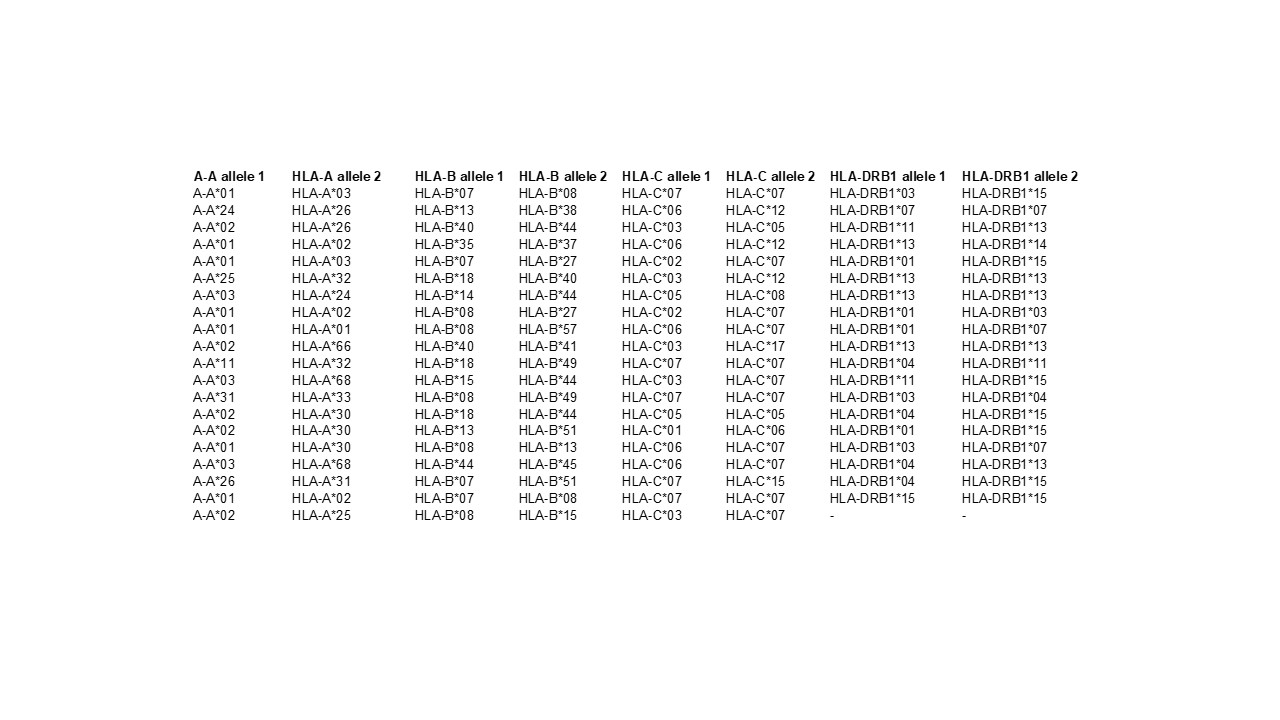

Supplement: Supplementary file 15 [file Image_15.jpeg]
